# Chromatin structure and DNA damage repair

**DOI:** 10.1186/1756-8935-1-9

**Published:** 2008-11-12

**Authors:** Christoffel Dinant, Adriaan B Houtsmuller, Wim Vermeulen

**Affiliations:** 1 Department of Pathology, Josephine Nefkens Institute, Erasmus MC, Rotterdam, the Netherlands; 2 Department of Cell Biology and Genetics, Erasmus MC, Dr. Molewaterplein 50, 3015 GE Rotterdam, the Netherlands

## Abstract

The integrity of the genome is continuously challenged by both endogenous and exogenous DNA damaging agents. These damaging agents can induce a wide variety of lesions in the DNA, such as double strand breaks, single strand breaks, oxidative lesions and pyrimidine dimers. The cell has evolved intricate DNA damage response mechanisms to counteract the genotoxic effects of these lesions. The two main features of the DNA damage response mechanisms are cell-cycle checkpoint activation and, at the heart of the response, DNA repair. For both damage signalling and repair, chromatin remodelling is most likely a prerequisite. Here, we discuss current knowledge on chromatin remodelling with respect to the cellular response to DNA damage, with emphasis on the response to lesions resolved by nucleotide excision repair. We will discuss the role of histone modifications as well as their displacement or exchange in nucleotide excision repair and make a comparison with their requirement in transcription and double strand break repair.

## Introduction

Proper functioning of all living organisms depends on faithful maintenance of genomic information. Although it is generally believed that information stored is relatively safe and stable, the integrity of DNA is continuously challenged by numerous genotoxic agents and environmental stress. Essential cellular functions such as oxidative respiration and lipid peroxidation create reactive oxygen species that can damage DNA. In addition, spontaneous hydrolysis of nucleotides induces non-instructive abasic sites. Finally, environmental physical and chemical agents, such as ultraviolet (UV) and ionising radiation, as well as numerous genotoxic chemicals present in food or combustion products in the air, induce a wide variety of DNA lesions. It has been estimated that in an average mammalian cell ten to a hundred thousand DNA lesions are introduced each day [1].

The consequences of DNA damage are diverse and adverse. Acute cellular effects arise from impeded gene transcription and DNA replication, causing cellular malfunctioning, irreversible cell cycle arrest (senescence) or cell death (apoptosis) which are important factors in (premature) aging [2,3]. DNA lesions interfere with proper chromosome segregation during cell division resulting in chromosome aberrations. In addition, replication errors due to DNA damage may introduce irreversible mutations. Chromosomal aberrations as well as mutations in coding genes may lead to carcinogenesis [3].

To counteract the severe biological consequences of DNA lesions an intricate network of genome surveillance mechanisms or DNA damage response (DDR) processes has evolved. The heart of this defence system is formed by complementary DNA repair systems that cover most of the genetic insults. In addition to the direct removal of lesions, DNA injuries trigger a signalling cascade that results in a slowdown of cell cycle progression, providing cells more time to repair DNA damage prior to replication or cell division.

The template for the DDR, damaged DNA, is packed into chromatin and it is expected that, analogous to other chromatin-associated processes such as replication and transcription [4,5], chromatin structure influences the DDR and *vice versa*. Recently, an array of different types of chromatin structural modulations has been reported in relation to the DDR. In this review we summarize and discuss current knowledge on chromatin remodelling, with emphasis on one specific DNA repair process, nucleotide excision repair (NER). For recent reviews on the connection between chromatin remodelling and repair pathways other than NER, see [6-14].

### DNA repair

The many different types of DNA lesions cannot be repaired by a single repair system. Instead, a number of specific repair processes have evolved that each remove a subset of lesions [15,16]. At least four major damage repair pathways operate in mammals: NER, base excision repair (BER), homologous recombination (HR) and end joining (EJ). NER deals with the wide class of single-strand lesions that destabilize the double helix, potentially obstructing transcription and replication. Small types of chemical alterations in the bases and single-strand DNA breaks are targeted by BER. Lesions for NER and BER affect only one of the strands of the double helix. In both processes the injury is excised and the resulting gap is filled by DNA synthesis using the intact complementary strand as a template, enabling error-free DNA repair. To properly heal the more problematic double-strand breaks (DSBs) two major pathways have developed. In mammals HR appears to be the predominant mode of DSB repair in S and G2, when an intact second copy of the sequence (sister chromatid) is available. The more error-prone non-homologous EJ operates mainly in G1 phase, but can also work on DSBs in S phase.

**Figure 1:**
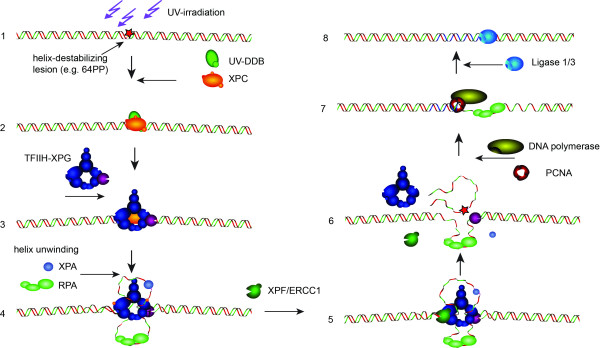
**Mammalian nucleotide excision repair mechanism**. Schematic representation of the mammalian GG-NER process subdivided into different steps. The TC-NER pathway only differs in the mode of detection, which occurs by lesion-stalled RNA polymerase II, and is omitted for simplicity. 1. DNA helix locally disturbed by a (e.g. UV-induced) NER-inducing lesion (indicated by a red star). 2. Binding of the two GG-NER-specific damage recognition complexes UV-DDB and XPC/HR23B/Cen2. 3. Lesion-bound XPC is a substrate for TFIIH and XPG. 4. The helicase activity of TFIIH (requiring ATP-hydrolysis) increases the local unwinding. This structure is stabilised by binding of XPA (damaged strand) and RPA (covers the opposite non-damaged strand). Likely, at his stage XPC is released. 5. The structure-specific nuclease XPF/ERCC1 binds the pre-incision complex. 6. XPG and XPF-ERCC1 incise 3’ and 5’ of the lesion, respectively, thereby releasing a stretch of 25-30 nucleotides including the lesion, after which most pre-incision factors release. RPA and XPG are thought to help loading of the (7.) replication factors, PCNA and either DNA polymerase δ or ε, that fill in the ss-gap. 8. The reaction is completed by the sealing activity of either ligase 1 or the complex XRCC1/Ligase 3.

NER is a versatile repair pathway able to remove many different types of single-strand lesions including the major UV-induced DNA photoproducts: cyclobutane pyrimidine dimers and 6-4 photoproducts, as well as large or bulky chemical adducts [17]. In actively transcribed genes, such lesions cause stalling of RNA polymerase II, which in turn recruits downstream NER proteins, a pathway termed transcription-coupled NER (TC-NER) [18]. In regions of the genome that are not actively transcribed, helix-distorting lesions are detected by the collective action of the UV-damaged DNA-binding protein (UV-DDB) complex and the xeroderma pigmentosum group C (XPC)-containing complex, initiating the global genome NER (GG-NER) sub-pathway [17]. Both UV-DDB and XPC complexes are able to bind to a surprisingly broad range of lesions that create short stretches of unpaired bases. The substrate versatility of the XPC complex is achieved by binding to the unpaired bases in the non-damaged strand opposite the lesion [19,20]. After recognition of the damage by either stalled RNA polymerase II or the XPC complex, repair complexes are assembled from freely diffusing NER factors (Figure 1, showing only the GG-NER pathway). The two helicases (XPB and XPD as part of the multi-functional, multi-subunit repair/transcription factor TFIIH), further unwind the helix after which two single-strand DNA-binding proteins (XPA and RPA) stabilize the unwound structure and properly orient the two structure-specific endonucleases (XPF-ERCC1 and XPG) that incise at some distance at either site of the lesion. The resulting 25 to 30 nucleotide single-strand gap is filled by the DNA replication machinery and finally sealed by one of the two DNA ligases (Ligase1 and XRCC1-Lig3) [16,21-24].

#### Cell-cycle checkpoint activation

To allow a cell to repair DNA lesions before it replicates over lesions that reduce the fidelity of polymerases (causing mutations) or passes through mitosis and propagates potentially harmful mutations to the daughter cells, DNA damage checkpoints temporarily block the cell cycle in G1, S or G2 phases in response to genotoxic stress. The three mammalian phosphoinositide 3-kinase-like kinases (PIKKs) ATM, ATR and DNA-PKcs have a central role in the activation of DNA damage checkpoints. These kinases induce a cascade of phosphorylations on a large number of different substrates, via mediators and transducers to effector molecules, such as the checkpoint kinases Chk1 and Chk2 [25-28]. Other targets include the histone H2A variant H2AX, checkpoint mediator protein 53BP1, DSB recognition factor NBS1 and many others [29,30]. ATM, ATR and DNA-PKcs are not only required for activating cell-cycle checkpoints but they also phosphorylate many substrates involved in other aspects of the DDR.

Although most types of DNA lesion have the ability to trigger cell-cycle checkpoint activation, damage signalling has been mainly studied in relation to DSBs. DNA DSBs induced by ionizing radiation generally cause large-scale chromatin rearrangements, initiated by ATM-dependent phosphorylation of H2AX (see below) [31]. Phosphorylated H2AX (γH2AX) triggers the accumulation of a multitude of different DSB repair and DNA damage signalling molecules, thereby concentrating repair proteins in small discrete nuclear foci termed ionizing radiation-induced foci (IRIF). Although their nature and function has been a topic of debate, it is believed that they at least play a role in the signalling pathway. Furthermore, it has been proposed that a function of increased local concentrations of proteins involved in the DDR will both stimulate repair and serve as an amplification of checkpoint signals [32,33]. As even a single endonuclease-induced DSB can induce chromosomal instabilities [34], amplification of checkpoint signals is most likely required to block the cell cycle until the DSB is repaired.

Proteins involved in NER typically bind to areas of DNA damage resulting in temporary immobilization of the proteins, but rather than accumulating in foci, they retain a homogenous distribution in G1 and G2 [35,36]. This also suggests that signal amplification by increased protein concentrations through foci formation, whether for activation of a cell-cycle checkpoint or for more efficient repair, does not play a role in NER. In addition, there is no evidence that NER-inducing lesions directly activate one of the PIKKs. However, during S-phase IRIF-like foci are formed when NER lesions generate stalled DNA replication forks, which create single-stranded stretches that are quickly covered by RPA/ATRIP and finally activate ATR, triggering replication-stress signalling [37,38]. Recently however, evidence has been provided that NER intermediates (short single-strand gaps, resulting from excised lesions) also activate ATR [39,40] outside S phase. It is currently unknown whether NER-induced ATR-signalling requires amplification in another way than by formation of foci as seen in DSB repair.

#### Chromatin remodelling

**Figure 2:**
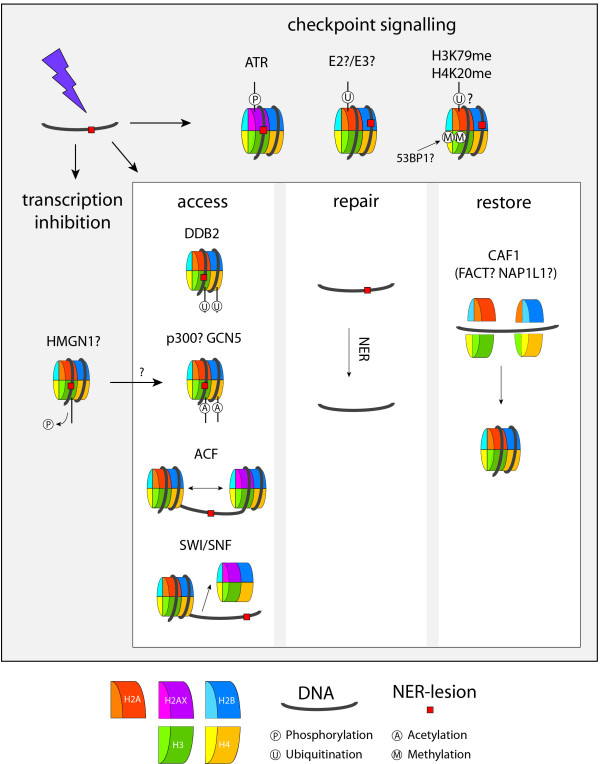
**Chromatin modifications and their consequences in response to lesions that are repaired by NER**. Induction of NER-lesions (by e.g. UV-light), results in three chromatin-associated responses: checkpoint signalling, transcription inhibition and DNA repair, of which the latter includes the postulated “access, repair, restore” model [133]. Checkpoint signalling involves phosphorylation of H2AX by ATR, ubiquitination of H2A (H2A-Ub) and methylated lysine residues H3K79 and H4K20. H2A-Ub and H3K79me/H4K20me are likely involved in checkpoint signalling upon NER activation, analogous to the DSB-response where H2A ubiquitination by RNF8 is required for the binding of checkpoint protein 53BP1 to H3K79me/H4K20me. Dephosphorylation of residues S10 and T11 of H3, possibly involving HMGN1, might contribute to transcription inhibition upon NER-activation and allow acetylation of H3K9, suggested to induce an open chromatin conformation enhancing access of the repair machinery to DNA. Other chromatin remodelling events promoting access to DNA repair proteins are ubiquitination of H3 and H4 by DDB2, acetylation of H3K14 and H4 by GCN5 and possibly p300, nucleosome sliding by ACF and nucleosome removal by SWI/SNF. After the lesion has been repaired, reincorporation of histones by CAF1 and possibly FACT or NAP1L1 restores the chromatin to its pre-damage conformation. Question marks indicate speculative activities.

In transcription and replication, changes in the chromatin structure are required in order to allow binding of the factors involved [4,5]. There is increasing evidence that this is also the case for DNA repair. Chromatin remodelling to alter the accessibility of proteins to DNA occurs by two mechanisms: covalent histone modifications by means of post-translational modifications (PTMs) and displacement of histones or entire nucleosomes, either by sliding along the DNA or by removal. In this review we will discuss recent literature addressing questions of if and how the DDR, and more particularly NER, requires modifications of chromatin structure and whether sequence-specific epigenetic marks are restored after repair. Figure 2 presents a model of known and speculated NER-associated chromatin modifications and the factors that are involved. In addition, in Tables 1 and 2 NER-related chromatin remodelling events are summarized and compared with these events during transcription.

**Table 1:** Histone modifications associated with transcription and nucleotide excision repair.

PTM	Transcription	NER
H2AXS139ph/yH2AS129ph	**?**	**+**
yH2AS122ph	**?**	**-**
H2Aub	**-**	**+**
H3K9/K14ac	**+**	**+**
H3K79me	**+**	**+**
H3S10ph	**+**	**-**
H3T11ph	**+**	**-**
H4K20me	**-**	**+**
H3/H4ub	**?**	**+**

ph = phosphorylation; ac = acetylation; me = methylation; ub = ubiquitination, '+' = upregulation or positive effect of the post-translational modification (PTM), '-' = downregulation or negative effect of the PTM and '?' = unknown function.

**Table 2:** Histone displacement associated with transcription and nucleotide excision repair.

**Protein**	**Transcription**	**NER**
**ATP-dependent motor proteins**		
ACF	**-**	**+**
SWI/SNF	**+**	**+**
CSB	**+**	**+**
**Histone Chaperones**		
CAF-1	**-**	**+**
NAP1L1	**+**	**?**
FACT	**+**	**?**

´+´ = remodeler enhances transcription or nucleotide excision repair (NER); ´-´ = remodeler is  involved in transcription repression and ´?´ = unknown function.

### Histone modifications

Covalent histone modifications or epigenetic changes are important regulatory elements for many biological processes. They function by influencing chromatin contacts through structural histone changes or influencing electrostatic interactions, and by recruiting non-histone proteins to chromatin [41]. Some covalent histone modifications that are involved in transcription are also associated with repair. On the other hand, modifications such as phosphorylation of H2AX appear to be unique to the DDR. In the following sections we will focus on the four epigenetic marks that are implicated in the DDR: phosphorylation, acetylation, methylation and ubiquitination. For a comprehensive review comparing covalent histone modifications during the different repair processes, see [14].

#### Phosphorylation

The hallmark DDR-related epigenetic change is phosphorylation of H2AX. Upon DSB induction by ionizing radiation, histone H2AX in the vicinity of DSBs is phosphorylated by ATM and DNA-PKcs at Serine 139. H2AX is phosphorylated over surprisingly long stretches of DNA of up to 2 × 10^6 ^bp around the break [8,31,42,43], creating a robust chromatin mark. In DSB repair, γH2AX is important for the formation of IRIF. In the absence of γH2AX, repair factors such as NBS1 (part of the homologous recombination-essential complex MRN) and Brca1 as well as checkpoint proteins MDC1 and 53BP1 fail to accumulate in IRIF, although they are still recruited to DNA damage [33]. Despite the dramatic chromatin changes and highly concentrated repair factors induced by γH2AX, its absence only mildly affects DSB repair [44]. This indicates that γH2AX-induced concentration of repair factors at sites of DNA damage does not play a crucial role in DNA repair. However, the formation of these IRIF through phosphorylation of H2AX is important for the activation of cell-cycle checkpoints in response to low doses of ionizing radiation [32], whereas γH2AX is dispensable for checkpoint activation at higher doses [44].

H2AX is also phosphorylated at residue S139 in response to UV irradiation [39,45-47], as well as its equivalent serine (S129 of H2A) in *S. cerevisiae *[48]. However, a S129A mutant strain was only slightly UV sensitive. Rather, it appeared that three different serine residues, S2, S18 and S122 play important roles in survival under UV. The serine 122 residue is involved in a general response to DNA damage, as it is required for HR, non-homologous EJ and NER. Surprisingly, this residue becomes dephosphorylated rather than phosphorylated upon UV irradiation, whereas other damage-inducing agents cause an increase of phosphorylation of S122 [48]. Whereas the DDR involves differential phosphorylation of multiple residues on both termini of H2A in yeast, no evidence is currently available that residues other than S139 of mammalian H2AX and H2A are implicated in the DDR.

Two histone residues of H3, serine 10 and threonine 11, appear to be a target of differential phosphorylation during NER [49,50]. Phosphorylation of both these residues is associated with transcription activation [51,52]. Like H2AS122 in yeast, H3S10 and H3T11 in mouse are dephosphorylated by UV irradiation and rephosphorylated after repair of the damage [49,50]. The function of this H3 modification in the DDR is not yet known. It is however tempting to speculate that the non-histone chromosomal protein HMGN1 plays a role, since HMGN1 inhibits the phosphorylation of H3S10 [53,54], enhances repair of UV lesions [53,54] and was shown to be recruited to TC-NER complexes [18]. A possible scenario for the role of HMGN1 in differential H3 phosphorylation within the DDR could be that HMGN1 is responsible for the dephosphorylation of H3S10. Hypophosphorylation of H3 at S10 and T11 is associated with transcription repression, and this might be one of the different mechanisms cells employ to inhibit transcription at UV-damaged areas. Dephosphorylation of S10 and T11 of H3 might also facilitate the acetylation of H3K9 (see below), the residue directly next to S10, by removing steric hindrance or neutralizing the negatively charged environment for an H3K9 acetyl transferase.

#### Acetylation

Another abundant histone PTM is differential acetylation, mainly associated with transcription activation. Different lysines in both histones H3 and H4 are targets for this modification, which neutralizes the basic charge of the lysine, thereby potentially altering the interaction between adjacent histones and between histones and DNA [41]. It was shown 20 years ago that histones become hyperacetylated in response to UV irradiation and that DNA repair is more efficient in hyperacetylated nucleosomes [55,56]. This suggests that changes in chromatin structure induced by acetylation make DNA more accessible not only for transcription factors but also for DNA repair activities.

Two histone acetyl transferases (HATs), Gcn5 and p300, responsible for the acetylation of multiple lysine residues within all four core histones, are implicated in the UV-induced DDR. In yeast, Gcn5 hyperacetylates H3 (at K9 and K14) at the repressed *MFA2 *promoter upon UV irradiation [57]. This acetylation is accompanied by increased accessibility of the DNA template (as tested by activity of restriction enzymes at the promoter), suggesting that the function for this modification is to allow access of proteins to the damaged DNA. Histone H3 acetylation by Gcn5 is implicated in regulating gene expression of ~5% of the yeast genome, including at *MFA2*. Another promoter, *RPB2*, which does not require Gcn5 for histone acetylation, also does not require Gcn5 for more efficient damage removal [58]. In mammalian cells, there is also evidence for the involvement of Gcn5 and the acetylation of H3 and H4 in the DDR. Gcn5 is recruited to DNA damage as part of a large complex, TFTC, which also includes SAP130, a splicing factor with sequence homology to DDB1, which is one of the subunits of the UV-DDB complex. Gcn5 is also part of another complex, STAGA, which interacts with both SAP130 and DDB1 in HeLa [59]. In yeast, the Rad16/Rad7 complex is implicated in UV-induced histone acetylation [60]. This protein complex is essential for yeast GG-NER [61,62]. Although GG-NER is conserved to mammals, surprisingly no sequence homologues for Rad16 or Rad7 have been found in mammalian cells. However, it has been suggested that the UV-DDB complex (containing DDB1 and the GG-NER-specific DDB2) might be a functional homologue of Rad16/Rad7 [63]. Besides the involvement of both these complexes in histone acetylation upon UV irradiation, there is more evidence pointing towards at least a partial functional homology between these complexes. For example, both are also involved in early steps of GG-NER and the ubiquitination of the NER recognition factor XPC/Rad4 [64,65].

Gcn5 regulates a subset of genes, whereas p300 HAT is a more global regulator of transcription [66]. Multiple proteins have been implicated in targeting p300 to UV damage, including Ing1B [67], DDB1 [68], PCNA [69], CSB [18] and p53 [70]. This suggests that besides Gcn5, p300 also has a function in acetylating histones before, during or after NER, although no direct evidence has so far been presented that p300 activity is required for efficient NER.

#### Methylation

A third abundant epigenetic histone mark is the differential methylation of lysine and arginine residues. Histones can be either mono-, di- or trimethylated and can either be a marker for transcriptionally active or inactive chromatin, depending on the type of methylation and the residue involved. Arginine methylation is less well studied than lysine methylation and no connection of this modification with a DDR has been found so far. Methylation of lysines H3K4, H3K36 and H3K79 is associated with transcription activation, while methylation of H3K9 and H3K27 and H4K20 is connected to transcription repression [41]. In response to UV irradiation, two apparently antagonizing histone methylations, H3K79me and H4K20me, have been identified.

Studies in *S. cerevisiae *have shown that H3K79 methylation by Dot1p is required for efficient UV-damage response. Disruption of H3K79 methylation, by *dot1 *or *K79E *mutations, results in hypersensitivity to UV and intra-S phase checkpoint deficiency [71,72]. In a similar study, H4K20 and its methyltransferase Set9 were mutated, resulting in UV sensitivity and checkpoint deficiency in fission yeast [73]. Both H3K79 and H4K20 methylation are involved in checkpoint signalling after DSB induction by recruiting 53BP1/CRB2 to IRIF [73-77]. In addition to DSB, there are indications that 53BP1 is also activated by UV irradiation [29,78-80]. This indicates that histone methylation might have the same checkpoint activation function after UV damage induction as it has in response to DSBs.

#### Ubiquitination

The largest PTM of histones is by conjugation of ubiquitin or ubiquitin-like moieties to lysine residues. Ubiquitin is a small 8.5 kDa peptide that can either target the conjugated protein to proteasomal degradation or serve as a modifier for protein function [81,82]. Modification of proteins by ubiquitin usually occurs via a three-step enzymatic reaction, involving ubiquitin-activating, -conjugating and -ligating (respectively E1, E2 and E3) enzymes that conjugate either one (monoubiquitination) or multiple (polyubiquitination) ubiquitin moieties on target polypeptides, depending on the substrate-specific combination of used E2 and E3. All four core histones are targets for ubiquitination but the precise function for most histone ubiquitination activities remains obscure. During transcription, ubiquitination of H2A is generally associated with gene silencing whereas ubiquitination of H2B has been related to both gene activation and silencing [83-86]. Ubiquitination of H3 and H4 is less abundant and as yet no functional consequence has been assigned to this modification.

In yeast, histone H2B ubiquitination at lysine 123 by the Rad6/Bre1 E2/E3 complex is required in the response to several DNA damage sources, including UV [72,87]. Absence of this modification (either by mutating Rad6 or H2B (K123R)), affects activation of the checkpoint kinase Rad53 and appears connected with exposure of lysine 79-methylated H3 and its subsequent activation of another checkpoint protein Rad9 [71,72]. No evidence has yet been presented that ubiquitination of H2B is increased after DNA damage induction. In mammalian cells, H2A rather than H2B appears to be the main target of ubiquitination in response to UV irradiation [88]. Remarkably, this modification is not required for NER, but rather occurs as a consequence of functional NER, as several defined NER mutants did not result in UV-induced H2A ubiquitination. Damage-induced H2A ubiquitination has been further shown to depend on ATR, which suggests that it has a function in cell cycle signalling.

UV irradiation not only causes an increase of H2A ubiquitination, but also induces a temporary H3 and H4 ubiquitination [89]. A complex containing UV-DDB and CUL4A was required for this ubiquitination. H3 and H4 ubiquitination occurs early in the DDR, in contrast to H2A ubiquitination. Together with the notion that ubiquitinated H3 and H4 reduce nucleosomal stability, it has been suggested that by this ubiquitination the UV-DDB complex creates a chromatin environment that facilitates the assembly of the NER complex on damaged DNA [90].

Besides UV-induced H2A ubiquitination, ubiquitination of H2A and H2AX in response to DSB induction has also been recently observed [91-93]. This epigenetic change is dependent on the ubiquitin-conjugating (E2) enzyme Ubc13 and the E3-ligase RING finger-containing protein RNF8 [91-93]. The ubiquitination was shown to depend on H2AX phosphorylation and the subsequent recruitment and activation of MDC1, to which RNF8 binds. Ubiquitinated targets (H2A and H2AX) are crucial for the formation of IRIF and assembly of downstream repair and checkpoint factors (RAP80, BRCA1 and 53BP1). The dynamic equilibrium of differential H2A ubiquitination was shown to be important for genome stability as a mutant form of the H2A-specific deubiquitination (or DUB) enzyme USP3 causes delay of S phase progression and activation of checkpoints [94].

### Histone displacement and exchange

### ATP-dependent chromatin remodelling

Besides covalent histone modifications, another important mechanism that changes chromatin structure is accomplished by a series of ATP-dependent chromatin remodelling complexes. ATP-dependent chromatin remodelling involves displacement of histones, either by completely removing or replacing them, or by sliding whole nucleosomes along the DNA strand. Like covalent histone modifications, this activity can control the recruitment of DNA-interacting proteins to chromatin and it is a versatile control mechanism in all nuclear processes [95].

Next to a clear function in transcription regulation, a number of ATP-dependent remodellers have been shown to play a role during DSB repair, including SWR1, RSC, INO80, Rad54 and SWI/SNF [96-99]. Of these, only SWI/SNF has also been associated with NER in *in vitro *experiments [100]. All ATP-dependent chromatin remodelling complexes contain a motor subunit belonging to Snf2-like family of ATPases [101]. This Snf2-like family includes a few known DNA repair proteins such as Rad16 and Rad5 in yeast (no mammalian orthologues have been identified) and Rad54 and Rad26 (RAD54 and CSB in mammals, respectively). Within an *in vitro *accessibility assay the TC-NER-specific CSB disarranged regularly spaced nucleosomes in an ATP-dependent manner [102]. Furthermore CSB directly interacted with double-stranded DNA and histone tails, which were required for this activity, suggesting that CSB may act as a TC-NER chromatin remodeller. CSB was also found to stimulate transcription by its interaction with RNA polymerase II, providing yet another link between DNA repair and transcription [103]. The yeast Rad16, which harbours a Snf2-like domain, is exclusively involved in GG-NER [61], although no evidence exists for a role in chromatin remodelling. Rather, it acts in complex with Rad7 and Abf1 to generate superhelical torsion in DNA, and its activity appears to be hindered by intact nucleosomes [62,104].

Two ATP-dependent chromatin remodellers that have been shown to stimulate NER by their remodelling activity are ACF and SWI/SNF [100,105]. ACF is known to have a function in replication, especially of heterochromatin regions [106]. It also enhances binding of heterochromatin protein 1 (HP1) to transcriptionally inactive chromatin and it does not co-localize with RNA polymerase II at salivary gland polytene chromosomes in Drosophila. This suggests that ACF is associated with transcription repression rather than activation [107,108]. In contrast, SWI/SNF is mainly associated with transcription activation [109,110].

ACF consists of two subunits, Acf1 and ISWI, which move nucleosomes along the DNA, generating internucleosomal spaces of 50 to 60 base pairs, without removing histones [111]. This nucleosomal sliding enhances NER activity, mainly in the linker regions between nucleosomes [105]. It will be interesting to investigate whether the mammalian orthologue of ACF is also implicated in the DDR. In contrast, the yeast SWI/SNF complex rather enhances NER of lesions located in nucleosome core regions [100]. This *in vitro *remodelling activity by SWI/SNF is dependent on the presence of NER factors XPC, XPA and RPA. In addition, two subunits of the yeast SWI/SNF complex, Snf5 and Snf6, co-purified with the NER factors Rad4 (the yeast homologue of XPC) and Rad23 [112]. Furthermore, Snf5 and Snf6 were shown to enhance NER and rearrange chromatin at the silent *HML *locus after UV irradiation. Most likely, yeast SWI/SNF is recruited to DNA lesions by binding to the Rad4-Rad23 complex, which is an early event in NER.

### Histone chaperones

Genome function depends for a large part on the accessibility of the DNA template. Above, several mechanisms are summarized that provide more plasticity to the dense chromatin structure. However, long-range transactions on the DNA helix require more than simply increasing accessibility. During transcription, elongation and replication, polymerases progress over long distances on DNA and extended nucleoprotein filaments (involving RPA and RAD51) are formed in homologous recombination, which involves large-scale nucleosomal rearrangements. Although it is likely that the discussed chromatin remodellers provide sufficient space to allow these elongations, displaced nucleosomes need to be repositioned after termination of these reactions. While some ATP-dependent chromatin remodellers are able to (re)deposit histones onto DNA, it is likely that for these more robust chromatin changes specialized activities exist to restore the chromatin structure. These specialized enzymes are referred to as histone chaperones. Histone chaperones deposit core histones onto DNA in an ATP-independent manner [113].

ASF1 is a histone chaperone that works together with either CAF1 or HIRA to deposit H3/H4 dimers or tetramers. Throughout the cell cycle, ASF1-HIRA is responsible for the incorporation of H3 and H4, whereas ASF1-CAF1 is involved in replication-dependent histone deposition [114-116]. Upon UV damage induction, ASF1 promotes nucleosome assembly together with CAF1 in a NER-dependent manner [117-119]. CAF1 knockdown does not inhibit NER in mammalian cells, suggesting that this H3.1 deposition is part of a chromatin restoration step after damage has been repaired which likely has no or limited influence on the repair rate itself.

In yeast, ASF1 and CAF1 are also involved in the response to UV irradiation. Both *cac1 *(the yeast CAF1 gene) and *asf1 *mutants are sensitive to UV, but a *cac1 asf1 *double mutant is more sensitive than either single mutant [120,121]. This suggests that Asf1 can perform its function in the absence of CAF1 and vice versa, albeit at a lower efficiency.

So far, no evidence has been found for the involvement of an H2A/H2B histone chaperone in NER. Possible candidates for this function are NAP1L1 or FACT, responsible for H2A/H2B deposition during replication and transcription [122-124]. In fact, FACT was recently shown to co-purify in complex with H2AX, DNA-PK and PARP1 and to promote the integration and dissociation of H2AX in a reconstituted nucleosomes experiment [124]. Phosphorylation of H2AX by DNA-PK increased the exchange of H2AX, indicating that FACT might function in histone exchange during DNA repair.

## Discussion

Because factors involved in both DNA repair and transcription require access to DNA, it is not surprising that a number of remodelling proteins and histone modifications that are associated with active transcription are shared with NER (for example, SWI/SNF and H3 acetylation) (see Tables 1 and 2). However, in transcriptionally active chromatin, transcription over a damaged template should be prevented while repair factors should still be allowed to bind the damaged DNA. Upon UV damage induction, RNA synthesis is inhibited by several mechanisms to protect the cell against the production of potentially dangerous proteins or RNAs [125-127]. One of these mechanisms involves phosphorylation, ubiquitination and proteolytic breakdown of RNA polymerase II [128,129]. Physical removal of RNA polymerase II ensures that transcription does not take place at UV-damaged areas. In a more speculative scenario, a similar chromatin-mediated signal transduction pathway as for cell cycle control, through for example covalent histone modifications and histone displacement, can be envisaged to inhibit transcription in a compromised genome. Although this mode of damage-induced transcriptional control after genomic insult was proposed many years ago, evidence of whether this actually occurs is currently lacking.

It is unlikely that histone modifications and remodelling proteins with functions in preventing access to DNA during transcription inhibition have the opposite function during repair. Therefore, it is surprising to note that a number of chromatin-modification factors linked to NER are associated with transcription inhibition rather than activation (H3 dephosphorylation, H4K20me, H2Aub, CAF1). This may be explained by auxiliary functions of remodelling proteins and histone modifications in transcription inhibition at damaged areas rather than a direct role in repair. Alternatively they may play a role in restoration of chromatin status after repair has taken place. Indeed CAF1 activity at NER sites has been suggested to restore chromatin status after repair is finished rather than enhance repair by its remodelling activity [117]. Similarly, UV-induced and ATR-dependent phosphorylation of H2AX and ubiquitination of H2A both require active NER [39,40,88] to elicit the histone PTMs, suggestive of a post-repair event.

We have discussed some of the chromatin remodelling activities that take place in association with NER in light of what is known for repair of DSBs. Within this process, remodelled chromatin, especially by ATM-induced phosphorylation and subsequent ubiquitination of H2AX, has been shown to be a major signal in checkpoint activation and amplification [32,91-93]. UV irradiation also induced phosphorylation of H2AX and ubiquitination of H2A both in an ATR-dependent fashion, most likely resulting in amplification of checkpoint signalling. Surprisingly and in contrast with DSBs, UV lesions require NER activity prior to activation of ATR signalling [40,45], histone ubiquitination [88] and finally phosphorylation of key checkpoint proteins CHK1 and p53 [130]. This absence of chromatin-associated damage signalling might in part explain the extreme predisposition for cancer in naturally occurring NER mutants (for example, in NER-deficient xeroderma pigmentosum patients) on top of severely attenuated damage removal in these patients' cells [3]. There are further interesting differences between chromatin remodelling responses upon DSB induction and NER activation. For example, in yeast, serine 122 of H2A becomes dephosphorylated upon UV irradiation, while after induction of other types of damage this residue is phosphorylated [48]. Interestingly, the checkpoint activator protein p53 also shows differential phosphorylation at a different residue in response to UV and to γ-irradiation [131,132]. Moreover, NER-induced chromatin changes do not occur at microscopically discernable sub-nuclear structures such as IRIF that play an important role in the DDR of DSBs. One of the main challenges in the field of DDR research will be to identify further differences and similarities between responses to different types of lesions and to more precisely determine their functions.

### Future directions

There is an obvious connection of the DDR with chromatin modifications. However, our current knowledge is based on studies which each focus on a separate DDR factor or different aspect of chromatin remodelling. In order to obtain better insight into the complex network of chromatin-associated DDR, a more systematic approach should be employed by combining genetic screening, transcriptional profiling, proteomic analysis and imaging approaches to study the spatio-temporal organisation of the entire DDR. Chromatin immunoprecipitation and mass spectrometry will give us further insight into chromatin components and their modifications in response to genomic insults.

## Competing interests

The authors declare that they have no competing interests.

## Authors' contributions

CD wrote the initial draft and ABH and WV edited the manuscript. All authors have read and approved the final manuscript.

## References

[B1] Lindahl T (1993). Instability and decay of the primary structure of DNA. Nature.

[B2] Hasty P, Campisi J, Hoeijmakers J, van Steeg H, Vijg J (2003). Aging and genome maintenance: lessons from the mouse?. Science.

[B3] Mitchell JR, Hoeijmakers JH, Niedernhofer LJ (2003). Divide and conquer: nucleotide excision repair battles cancer and ageing. Curr Opin Cell Biol.

[B4] Li B, Carey M, Workman JL (2007). The role of chromatin during transcription. Cell.

[B5] Groth A, Rocha W, Verreault A, Almouzni G (2007). Chromatin challenges during DNA replication and repair. Cell.

[B6] Osley MA, Tsukuda T, Nickoloff JA (2007). ATP-dependent chromatin remodeling factors and DNA damage repair. Mutat Res.

[B7] Ataian Y, Krebs JE (2006). Five repair pathways in one context: chromatin modification during DNA repair. Biochem Cell Biol.

[B8] Costelloe T, Fitzgerald J, Murphy NJ, Flaus A, Lowndes NF (2006). Chromatin modulation and the DNA damage response. Exp Cell Res.

[B9] Altaf M, Saksouk N, Cote J (2007). Histone modifications in response to DNA damage. Mutat Res.

[B10] van Attikum H, Gasser SM (2005). The histone code at DNA breaks: a guide to repair?. Nat Rev Mol Cell Biol.

[B11] Wong LY, Recht J, Laurent BC (2006). Chromatin remodeling and repair of DNA double-strand breaks. J Mol Histol.

[B12] Wurtele H, Verreault A (2006). Histone post-translational modifications and the response to DNA double-strand breaks. Curr Opin Cell Biol.

[B13] Caldecott KW (2007). Mammalian single-strand break repair: mechanisms and links with chromatin. DNA Repair (Amst).

[B14] Escargueil AE, Soares DG, Salvador M, Larsen AK, Henriques JA (2008). What histone code for DNA repair?. Mutat Res.

[B15] Essers J, Vermeulen W, Houtsmuller AB (2006). DNA damage repair: anytime, anywhere?. Curr Opin Cell Biol.

[B16] Hoeijmakers JH (2001). Genome maintenance mechanisms for preventing cancer. Nature.

[B17] Gillet LC, Scharer OD (2006). Molecular mechanisms of mammalian global genome nucleotide excision repair. Chem Rev.

[B18] Fousteri M, Vermeulen W, van Zeeland AA, Mullenders LH (2006). Cockayne syndrome A and B proteins differentially regulate recruitment of chromatin remodeling and repair factors to stalled RNA polymerase II in vivo. Mol Cell.

[B19] Maillard O, Solyom S, Naegeli H (2007). An aromatic sensor with aversion to damaged strands confers versatility to DNA repair. PLoS Biol.

[B20] Min JH, Pavletich NP (2007). Recognition of DNA damage by the Rad4 nucleotide excision repair protein. Nature.

[B21] Evans E, Moggs JG, Hwang JR, Egly JM, Wood RD (1997). Mechanism of open complex and dual incision formation by human nucleotide excision repair factors. EMBO J.

[B22] Moser J, Kool H, Giakzidis I, Caldecott K, Mullenders LH, Fousteri MI (2007). Sealing of chromosomal DNA nicks during nucleotide excision repair requires XRCC1 and DNA ligase III alpha in a cell-cycle-specific manner. Mol Cell.

[B23] de Laat WL, Jaspers NG, Hoeijmakers JH (1999). Molecular mechanism of nucleotide excision repair. Genes Dev.

[B24] Volker M, Moné MJ, Karmakar P, Hoffen A, Schul W, Vermeulen W, Hoeijmakers JHJ, van Driel R, Zeeland AA, Mullenders LHF (2001). Sequential assembly of the nucleotide excision repair factors in vivo. Mol Cell.

[B25] Falck J, Coates J, Jackson SP (2005). Conserved modes of recruitment of ATM, ATR and DNA-PKcs to sites of DNA damage. Nature.

[B26] Bartek J, Lukas J (2007). DNA damage checkpoints: from initiation to recovery or adaptation. Curr Opin Cell Biol.

[B27] Liu Q, Guntuku S, Cui XS, Matsuoka S, Cortez D, Tamai K, Luo G, Carattini-Rivera S, DeMayo F, Bradley A, Donehower LA, Elledge SJ (2000). Chk1 is an essential kinase that is regulated by Atr and required for the G(2)/M DNA damage checkpoint. Genes Dev.

[B28] Shiloh Y (2003). ATM and related protein kinases: safeguarding genome integrity. Nat Rev Cancer.

[B29] Jowsey P, Morrice NA, Hastie CJ, McLauchlan H, Toth R, Rouse J (2007). Characterisation of the sites of DNA damage-induced 53BP1 phosphorylation catalysed by ATM and ATR. DNA Repair (Amst).

[B30] Bakkenist CJ, Kastan MB (2004). Initiating cellular stress responses. Cell.

[B31] Rogakou EP, Pilch DR, Orr AH, Ivanova VS, Bonner WM (1998). DNA double-stranded breaks induce histone H2AX phosphorylation on serine 139. J Biol Chem.

[B32] Fernandez-Capetillo O, Chen HT, Celeste A, Ward I, Romanienko PJ, Morales JC, Naka K, Xia Z, Camerini-Otero RD, Motoyama N, Carpenter PB, Bonner WM, Chen J, Nussenzweig A (2002). DNA damage-induced G2-M checkpoint activation by histone H2AX and 53BP1. Nat Cell Biol.

[B33] Celeste A, Fernandez-Capetillo O, Kruhlak MJ, Pilch DR, Staudt DW, Lee A, Bonner RF, Bonner WM, Nussenzweig A (2003). Histone H2AX phosphorylation is dispensable for the initial recognition of DNA breaks. Nat Cell Biol.

[B34] Bennett CB, Lewis AL, Baldwin KK, Resnick MA (1993). Lethality induced by a single site-specific double-strand break in a dispensable yeast plasmid. Proc Natl Acad Sci USA.

[B35] Zotter A, Luijsterburg MS, Warmerdam DO, Ibrahim S, Nigg A, van Cappellen WA, Hoeijmakers JH, van Driel R, Vermeulen W, Houtsmuller AB (2006). Recruitment of the nucleotide excision repair endonuclease XPG to sites of UV-induced DNA damage depends on functional TFIIH. Mol Cell Biol.

[B36] Houtsmuller AB, Rademakers S, Nigg AL, Hoogstraten D, Hoeijmakers JH, Vermeulen W (1999). Action of DNA repair endonuclease ERCC1/XPF in living cells. Science.

[B37] Ward IM, Chen J (2001). Histone H2AX is phosphorylated in an ATR-dependent manner in response to replicational stress. J Biol Chem.

[B38] Jeggo P, Lobrich M (2006). Radiation-induced DNA damage responses. Radiat Prot Dosimetry.

[B39] Hanasoge S, Ljungman M (2007). H2AX phosphorylation after UV-irradiation is triggered by DNA repair intermediates and is mediated by the ATR kinase. Carcinogenesis.

[B40] Matsumoto M, Yaginuma K, Igarashi A, Imura M, Hasegawa M, Iwabuchi K, Date T, Mori T, Ishizaki K, Yamashita K, Inobe M, Matsunaga T (2007). Perturbed gap-filling synthesis in nucleotide excision repair causes histone H2AX phosphorylation in human quiescent cells. J Cell Sci.

[B41] Kouzarides T (2007). Chromatin modifications and their function. Cell.

[B42] Rogakou EP, Boon C, Redon C, Bonner WM (1999). Megabase chromatin domains involved in DNA double-strand breaks in vivo. J Cell Biol.

[B43] Lowndes NF, Toh GW (2005). DNA repair: the importance of phosphorylating histone H2AX. Curr Biol.

[B44] Celeste A, Petersen S, Romanienko PJ, Fernandez-Capetillo O, Chen HT, Sedelnikova OA, Reina-San-Martin B, Coppola V, Meffre E, Difilippantonio MJ, Redon C, Pilch DR, Olaru A, Eckhaus M, Camerini-Otero RD, Tessarollo L, Livak F, Manova K, Bonner WM, Nussenzweig MC, Nussenzweig A (2002). Genomic instability in mice lacking histone H2AX. Science.

[B45] O'Driscoll M, Ruiz-Perez VL, Woods CG, Jeggo PA, Goodship JA (2003). A splicing mutation affecting expression of ataxia-telangiectasia and Rad3-related protein (ATR) results in Seckel syndrome. Nat Genet.

[B46] Stiff T, Walker SA, Cerosaletti K, Goodarzi AA, Petermann E, Concannon P, O'Driscoll M, Jeggo PA (2006). ATR-dependent phosphorylation and activation of ATM in response to UV treatment or replication fork stalling. Embo J.

[B47] Marti TM, Hefner E, Feeney L, Natale V, Cleaver JE (2006). H2AX phosphorylation within the G1 phase after UV irradiation depends on nucleotide excision repair and not DNA double-strand breaks. Proc Natl Acad Sci USA.

[B48] Moore JD, Yazgan O, Ataian Y, Krebs JE (2006). Diverse roles for histone H2A modifications in DNA damage response pathways in yeast. Genetics.

[B49] Sen SP, De Benedetti A (2006). TLK1B promotes repair of UV-damaged DNA through chromatin remodeling by Asf1. BMC Mol Biol.

[B50] Shimada M, Niida H, Zineldeen DH, Tagami H, Tanaka M, Saito H, Nakanishi M (2008). Chk1 is a histone H3 threonine 11 kinase that regulates DNA damage-induced transcriptional repression. Cell.

[B51] Clements A, Poux AN, Lo WS, Pillus L, Berger SL, Marmorstein R (2003). Structural basis for histone and phosphohistone binding by the GCN5 histone acetyltransferase. Mol Cell.

[B52] Ivaldi MS, Karam CS, Corces VG (2007). Phosphorylation of histone H3 at Ser10 facilitates RNA polymerase II release from promoter-proximal pausing in Drosophila. Genes Dev.

[B53] Lim JH, Catez F, Birger Y, West KL, Prymakowska-Bosak M, Postnikov YV, Bustin M (2004). Chromosomal protein HMGN1 modulates histone H3 phosphorylation. Mol Cell.

[B54] Birger Y, West KL, Postnikov YV, Lim JH, Furusawa T, Wagner JP, Laufer CS, Kraemer KH, Bustin M (2003). Chromosomal protein HMGN1 enhances the rate of DNA repair in chromatin. Embo J.

[B55] Ramanathan B, Smerdon MJ (1986). Changes in nuclear protein acetylation in UV-damaged human cells. Carcinogenesis.

[B56] Ramanathan B, Smerdon MJ (1989). Enhanced DNA repair synthesis in hyperacetylated nucleosomes. J Biol Chem.

[B57] Yu Y, Teng Y, Liu H, Reed SH, Waters R (2005). UV irradiation stimulates histone acetylation and chromatin remodeling at a repressed yeast locus. Proc Natl Acad Sci USA.

[B58] Teng Y, Yu Y, Waters R (2002). The *Saccharomyces cerevisiae *histone acetyltransferase Gcn5 has a role in the photoreactivation and nucleotide excision repair of UV-induced cyclobutane pyrimidine dimers in the MFA2 gene. J Mol Biol.

[B59] Martinez E, Palhan VB, Tjernberg A, Lymar ES, Gamper AM, Kundu TK, Chait BT, Roeder RG (2001). Human STAGA complex is a chromatin-acetylating transcription coactivator that interacts with pre-mRNA splicing and DNA damage-binding factors in vivo. Mol Cell Biol.

[B60] Teng Y, Liu H, Gill HW, Yu Y, Waters R, Reed SH (2007). *Saccharomyces cerevisiae *Rad16 mediates ultraviolet-dependent histone H3 acetylation required for efficient global genome nucleotide-excision repair. EMBO Rep.

[B61] Verhage R, Zeeman AM, de Groot N, Gleig F, Bang DD, Putte P van de, Brouwer J (1994). The RAD7 and RAD16 genes, which are essential for pyrimidine dimer removal from the silent mating type loci, are also required for repair of the nontranscribed strand of an active gene in *Saccharomyces cerevisiae*. Mol Cell Biol.

[B62] Yu S, Owen-Hughes T, Friedberg EC, Waters R, Reed SH (2004). The yeast Rad7/Rad16/Abf1 complex generates superhelical torsion in DNA that is required for nucleotide excision repair. DNA Repair (Amst).

[B63] Lombaerts M, Peltola PH, Visse R, den Dulk H, Brandsma JA, Brouwer J (1999). Characterization of the rhp7(+) and rhp16(+) genes in *Schizosaccharomyces pombe*. Nucleic Acids Res.

[B64] Sugasawa K, Okuda Y, Saijo M, Nishi R, Matsuda N, Chu G, Mori T, Iwai S, Tanaka K, Tanaka K, Hanaoka F (2005). UV-induced ubiquitylation of XPC protein mediated by UV-DDB-ubiquitin ligase complex. Cell.

[B65] Gillette TG, Yu S, Zhou Z, Waters R, Johnston SA, Reed SH (2006). Distinct functions of the ubiquitin-proteasome pathway influence nucleotide excision repair. Embo J.

[B66] Sterner DE, Berger SL (2000). Acetylation of histones and transcription-related factors. Microbiol Mol Biol Rev.

[B67] Kuo WH, Wang Y, Wong RP, Campos EI, Li G (2007). The ING1b tumor suppressor facilitates nucleotide excision repair by promoting chromatin accessibility to XPA. Exp Cell Res.

[B68] Rapic-Otrin V, McLenigan MP, Bisi DC, Gonzalez M, Levine AS (2002). Sequential binding of UV DNA damage binding factor and degradation of the p48 subunit as early events after UV irradiation. Nucleic Acids Res.

[B69] Hasan S, Hassa PO, Imhof R, Hottiger MO (2001). Transcription coactivator p300 binds PCNA and may have a role in DNA repair synthesis. Nature.

[B70] Rubbi CP, Milner J (2003). p53 is a chromatin accessibility factor for nucleotide excision repair of DNA damage. Embo J.

[B71] Bostelman LJ, Keller AM, Albrecht AM, Arat A, Thompson JS (2007). Methylation of histone H3 lysine-79 by Dot1p plays multiple roles in the response to UV damage in *Saccharomyces cerevisiae*. DNA Repair (Amst).

[B72] Giannattasio M, Lazzaro F, Plevani P, Muzi-Falconi M (2005). The DNA damage checkpoint response requires histone H2B ubiquitination by Rad6-Bre1 and H3 methylation by Dot1. J Biol Chem.

[B73] Sanders SL, Portoso M, Mata J, Bahler J, Allshire RC, Kouzarides T (2004). Methylation of histone H4 lysine 20 controls recruitment of Crb2 to sites of DNA damage. Cell.

[B74] Botuyan MV, Lee J, Ward IM, Kim JE, Thompson JR, Chen J, Mer G (2006). Structural basis for the methylation state-specific recognition of histone H4-K20 by 53BP1 and Crb2 in DNA repair. Cell.

[B75] Kim J, Daniel J, Espejo A, Lake A, Krishna M, Xia L, Zhang Y, Bedford MT (2006). Tudor, MBT and chromo domains gauge the degree of lysine methylation. EMBO Rep.

[B76] Du LL, Nakamura TM, Russell P (2006). Histone modification-dependent and -independent pathways for recruitment of checkpoint protein Crb2 to double-strand breaks. Genes Dev.

[B77] Huyen Y, Zgheib O, Ditullio RA, Gorgoulis VG, Zacharatos P, Petty TJ, Sheston EA, Mellert HS, Stavridi ES, Halazonetis TD (2004). Methylated lysine 79 of histone H3 targets 53BP1 to DNA double-strand breaks. Nature.

[B78] Silverman J, Takai H, Buonomo SB, Eisenhaber F, de Lange T (2004). Human Rif1, ortholog of a yeast telomeric protein, is regulated by ATM and 53BP1 and functions in the S-phase checkpoint. Genes Dev.

[B79] Rappold I, Iwabuchi K, Date T, Chen J (2001). Tumor suppressor p53 binding protein 1 (53BP1) is involved in DNA damage-signaling pathways. J Cell Biol.

[B80] Anderson L, Henderson C, Adachi Y (2001). Phosphorylation and rapid relocalization of 53BP1 to nuclear foci upon DNA damage. Mol Cell Biol.

[B81] Herrmann J, Lerman LO, Lerman A (2007). Ubiquitin and ubiquitin-like proteins in protein regulation. Circ Res.

[B82] Glickman MH, Ciechanover A (2002). The ubiquitin-proteasome proteolytic pathway: destruction for the sake of construction. Physiol Rev.

[B83] Kao CF, Hillyer C, Tsukuda T, Henry K, Berger S, Osley MA (2004). Rad6 plays a role in transcriptional activation through ubiquitylation of histone H2B. Genes Dev.

[B84] Osley MA (2006). Regulation of histone H2A and H2B ubiquitylation. Brief Funct Genomic Proteomic.

[B85] de Napoles M, Mermoud JE, Wakao R, Tang YA, Endoh M, Appanah R, Nesterova TB, Silva J, Otte AP, Vidal M, Koseki H, Brockdorff N (2004). Polycomb group proteins Ring1A/B link ubiquitylation of histone H2A to heritable gene silencing and X inactivation. Dev Cell.

[B86] Turner SD, Ricci AR, Petropoulos H, Genereaux J, Skerjanc IS, Brandl CJ (2002). The E2 ubiquitin conjugase Rad6 is required for the ArgR/Mcm1 repression of ARG1 transcription. Mol Cell Biol.

[B87] Robzyk K, Recht J, Osley MA (2000). Rad6-dependent ubiquitination of histone H2B in yeast. Science.

[B88] Bergink S, Salomons FA, Hoogstraten D, Groothuis TA, de Waard H, Wu J, Yuan L, Citterio E, Houtsmuller AB, Neefjes J, Hoeijmakers JHJ, Vermeulen W, Dantuma NP (2006). DNA damage triggers nucleotide excision repair-dependent monoubiquitylation of histone H2A. Genes Dev.

[B89] Wang H, Zhai L, Xu J, Joo HY, Jackson S, Erdjument-Bromage H, Tempst P, Xiong Y, Zhang Y (2006). Histone H3 and H4 ubiquitylation by the CUL4-DDB-ROC1 ubiquitin ligase facilitates cellular response to DNA damage. Mol Cell.

[B90] Luijsterburg MS, Goedhart J, Moser J, Kool H, Geverts B, Houtsmuller AB, Mullenders LH, Vermeulen W, van Driel R (2007). Dynamic in vivo interaction of DDB2 E3 ubiquitin ligase with UV-damaged DNA is independent of damage-recognition protein XPC. J Cell Sci.

[B91] Mailand N, Bekker-Jensen S, Faustrup H, Melander F, Bartek J, Lukas C, Lukas J (2007). RNF8 Ubiquitylates Histones at DNA Double-Strand Breaks and Promotes Assembly of Repair Proteins. Cell.

[B92] Huen MS, Grant R, Manke I, Minn K, Yu X, Yaffe MB, Chen J (2007). RNF8 transduces the DNA-damage signal via histone ubiquitylation and checkpoint protein assembly. Cell.

[B93] Kolas NK, Chapman JR, Nakada S, Ylanko J, Chahwan R, Sweeney FD, Panier S, Mendez M, Wildenhain J, Thomson TM, Pelletier L, Jackson SP, Durocher D (2007). Orchestration of the DNA-damage response by the rnf8 ubiquitin ligase. Science.

[B94] Nicassio F, Corrado N, Vissers JH, Areces LB, Bergink S, Marteijn JA, Geverts B, Houtsmuller AB, Vermeulen W, Di Fiore PP, Citterio E (2007). Human USP3 is a chromatin modifier required for S phase progression and genome stability. Curr Biol.

[B95] Gangaraju VK, Bartholomew B (2007). Mechanisms of ATP dependent chromatin remodeling. Mutat Res.

[B96] Osley MA, Shen X (2006). Altering nucleosomes during DNA double-strand break repair in yeast. Trends Genet.

[B97] van Attikum H, Fritsch O, Hohn B, Gasser SM (2004). Recruitment of the INO80 complex by H2A phosphorylation links ATP-dependent chromatin remodeling with DNA double-strand break repair. Cell.

[B98] Morrison AJ, Highland J, Krogan NJ, Arbel-Eden A, Greenblatt JF, Haber JE, Shen X (2004). INO80 and gamma-H2AX interaction links ATP-dependent chromatin remodeling to DNA damage repair. Cell.

[B99] van Attikum H, Fritsch O, Gasser SM (2007). Distinct roles for SWR1 and INO80 chromatin remodeling complexes at chromosomal double-strand breaks. Embo J.

[B100] Hara R, Sancar A (2002). The SWI/SNF chromatin-remodeling factor stimulates repair by human excision nuclease in the mononucleosome core particle. Mol Cell Biol.

[B101] Lusser A, Kadonaga JT (2003). Chromatin remodeling by ATP-dependent molecular machines. Bioessays.

[B102] Citterio E, Boom V Van Den, Schnitzler G, Kanaar R, Bonte E, Kingston RE, Hoeijmakers JH, Vermeulen W (2000). ATP-Dependent chromatin remodeling by the Cockayne Syndrome B DNA repair-transcription-coupling factor. Mol Cell Biol.

[B103] Boom V van den, Citterio E, Hoogstraten D, Zotter A, Egly JM, van Cappellen WA, Hoeijmakers JH, Houtsmuller AB, Vermeulen W (2004). DNA damage stabilizes interaction of CSB with the transcription elongation machinery. J Cell Biol.

[B104] Li S, Ding B, Lejeune D, Ruggiero C, Chen X, Smerdon MJ (2007). The roles of Rad16 and Rad26 in repairing repressed and actively transcribed genes in yeast. DNA Repair (Amst).

[B105] Ura K, Araki M, Saeki H, Masutani C, Ito T, Iwai S, Mizukoshi T, Kaneda Y, Hanaoka F (2001). ATP-dependent chromatin remodeling facilitates nucleotide excision repair of UV-induced DNA lesions in synthetic dinucleosomes. Embo J.

[B106] Collins N, Poot RA, Kukimoto I, Garcia-Jimenez C, Dellaire G, Varga-Weisz PD (2002). An ACF1-ISWI chromatin-remodeling complex is required for DNA replication through heterochromatin. Nat Genet.

[B107] Deuring R, Fanti L, Armstrong JA, Sarte M, Papoulas O, Prestel M, Daubresse G, Verardo M, Moseley SL, Berloco M, Tsukiyama T, Wu C, Pimpinelli S, Tamkun JW (2000). The ISWI chromatin-remodeling protein is required for gene expression and the maintenance of higher order chromatin structure in vivo. Mol Cell.

[B108] Eskeland R, Eberharter A, Imhof A (2007). HP1 binding to chromatin methylated at H3K9 is enhanced by auxiliary factors. Mol Cell Biol.

[B109] Vignali M, Hassan AH, Neely KE, Workman JL (2000). ATP-dependent chromatin-remodeling complexes. Mol Cell Biol.

[B110] Kadam S, Emerson BM (2003). Transcriptional specificity of human SWI/SNF BRG1 and BRM chromatin remodeling complexes. Mol Cell.

[B111] Yang JG, Madrid TS, Sevastopoulos E, Narlikar GJ (2006). The chromatin-remodeling enzyme ACF is an ATP-dependent DNA length sensor that regulates nucleosome spacing. Nat Struct Mol Biol.

[B112] Gong F, Fahy D, Smerdon MJ (2006). Rad4-Rad23 interaction with SWI/SNF links ATP-dependent chromatin remodeling with nucleotide excision repair. Nat Struct Mol Biol.

[B113] Tyler JK (2002). Chromatin assembly. Cooperation between histone chaperones and ATP-dependent nucleosome remodeling machines. Eur J Biochem.

[B114] Polo SE, Almouzni G (2006). Chromatin assembly: a basic recipe with various flavours. Curr Opin Genet Dev.

[B115] Sharp JA, Fouts ET, Krawitz DC, Kaufman PD (2001). Yeast histone deposition protein Asf1p requires Hir proteins and PCNA for heterochromatic silencing. Curr Biol.

[B116] Green EM, Antczak AJ, Bailey AO, Franco AA, Wu KJ, Yates JR, Kaufman PD (2005). Replication-independent histone deposition by the HIR complex and Asf1. Curr Biol.

[B117] Polo SE, Roche D, Almouzni G (2006). New histone incorporation marks sites of UV repair in human cells. Cell.

[B118] Mello JA, Sillje HH, Roche DM, Kirschner DB, Nigg EA, Almouzni G (2002). Human Asf1 and CAF-1 interact and synergize in a repair-coupled nucleosome assembly pathway. EMBO Rep.

[B119] Green CM, Almouzni G (2003). Local action of the chromatin assembly factor CAF-1 at sites of nucleotide excision repair in vivo. Embo J.

[B120] Kaufman PD, Kobayashi R, Stillman B (1997). Ultraviolet radiation sensitivity and reduction of telomeric silencing in Saccharomyces cerevisiae cells lacking chromatin assembly factor-I. Genes Dev.

[B121] Tyler JK, Adams CR, Chen SR, Kobayashi R, Kamakaka RT, Kadonaga JT (1999). The RCAF complex mediates chromatin assembly during DNA replication and repair. Nature.

[B122] Reinberg D, Sims RJ (2006). de FACTo nucleosome dynamics. J Biol Chem.

[B123] Zlatanova J, Seebart C, Tomschik M (2007). Nap1: taking a closer look at a juggler protein of extraordinary skills. Faseb J.

[B124] Heo K, Kim H, Choi SH, Choi J, Kim K, Gu J, Lieber MR, Yang AS, An W (2008). FACT-mediated exchange of histone variant H2AX regulated by phosphorylation of H2AX and ADP-ribosylation of Spt16. Mol Cell.

[B125] Kantor GJ, Hull DR (1979). An effect of ultraviolet light on RNA and protein synthesis in nondividing human diploid fibroblasts. Biophys J.

[B126] Mone MJ, Volker M, Nikaido O, Mullenders LH, van Zeeland AA, Verschure PJ, Manders EM, van Driel R (2001). Local UV-induced DNA damage in cell nuclei results in local transcription inhibition. EMBO Rep.

[B127] Kruhlak M, Crouch EE, Orlov M, Montano C, Gorski SA, Nussenzweig A, Misteli T, Phair RD, Casellas R (2007). The ATM repair pathway inhibits RNA polymerase I transcription in response to chromosome breaks. Nature.

[B128] Rockx DA, Mason R, van Hoffen A, Barton MC, Citterio E, Bregman DB, van Zeeland AA, Vrieling H, Mullenders LH (2000). UV-induced inhibition of transcription involves repression of transcription initiation and phosphorylation of RNA polymerase II. Proc Natl Acad Sci USA.

[B129] Woudstra EC, Gilbert C, Fellows J, Jansen L, Brouwer J, Erdjument-Bromage H, Tempst P, Svejstrup JQ (2002). A Rad26-Def1 complex coordinates repair and RNA pol II proteolysis in response to DNA damage. Nature.

[B130] Mannhaupt G, Stucka R, Ehnle S, Vetter I, Feldmann H (1992). Molecular analysis of yeast chromosome II between *CMD1 *and *LYS2: *the excision repair gene *RAD16 *located in this region belongs to a novel group of finger proteins. Yeast.

[B131] Kapoor M, Lozano G (1998). Functional activation of p53 via phosphorylation following DNA damage by UV but not gamma radiation. Proc Natl Acad Sci USA.

[B132] Lu H, Taya Y, Ikeda M, Levine AJ (1998). Ultraviolet radiation, but not gamma radiation or etoposide-induced DNA damage, results in the phosphorylation of the murine p53 protein at serine-389. Proc Natl Acad Sci USA.

[B133] Smerdon MJ (1991). DNA repair and the role of chromatin structure. Curr Opin Cell Biol.

